# The protein transition: what determines the animal-to-plant (A:P) protein ratios in global diets

**DOI:** 10.3389/fnut.2025.1518793

**Published:** 2025-02-12

**Authors:** Adam Drewnowski, Kayla Hooker

**Affiliations:** ^1^Center for Public Health Nutrition, University of Washington, Seattle, WA, United States; ^2^Department of Nutrition and Exercise Physiology, Elson S. Floyd College of Medicine, Washington State University, Spokane, WA, United States

**Keywords:** protein transition, animal:plant protein ratios, FAO Food balance sheets, World Bank income classification, Bennett’s law, animal-based proteins, plant-based proteins, low and middle income countries (LMIC)

## Abstract

**Background:**

Several high-income countries have announced plans to reduce the animal-to-plant (A:P) protein ratios in their population diets. Their current A:P ratio is around 65:35, with two thirds of the protein coming from animal sources, meat, eggs, and dairy. Efforts to reduce the dietary A:P protein ratio to 50:50, 40:60, or below are sometimes referred to as a “healthy protein transition.”

**Methods:**

Analyses of Food and Agriculture Organization (FAO) and World Bank data were used to show that an opposing and far more important protein transition is taking place globally.

**Results:**

In most low- and middle-income countries (LMIC), the dietary A:P protein ratio was closely associated with, if not determined, by gross national incomes (GNI). As incomes rise, LMIC populations adopt more varied and more nutrient-rich diets with more animal proteins and especially meat. This protein transition, manifested by a strong observed relation between rising incomes and higher A:P protein ratios, follows a well-known principle of economics known as Bennett’s Law.

**Conclusion:**

Consumer education and regulatory and policy measures aimed at reducing dietary A:P protein ratios worldwide may not uncouple the fundamental relation between powerful economic forces and global diet structures.

## 1 Introduction

An important protein transition is currently occurring across lower-and middle-income countries (LMIC), mostly in the global south (1–3). Viewed as a key component of the broader and well-defined nutrition transition (4), the term protein transition refers to the replacement of traditional proteins from staple grains, pulses, and root crops with more animal-source foods and especially meat (5). This protein transition is largely driven by economic development and rising incomes, though the selection of specific animal protein: beef, pork, chicken, or dairy can vary across geographic regions, depending on tradition and cultures (1, 5). The LMIC trend toward *more* animal protein has been actively promoted by multiple actors (6, 7), including international agencies aiming to improve LMIC diet quality and population health.

Efforts by some high-income countries (HIC) to promote diets with *less* animal protein have been referred to as a “healthy protein transition” (8). Built around consumer education, technological advances, and policy measures (2), such efforts aim to replace meat and dairy with more plant-based options. The “healthy protein transition” has been vigorously promoted by researchers (2), foundations (9, 10) and by national and local government (11–13), aiming to stem climate change and also improve HIC diet quality and population health.

Mean protein consumption in the highest-income countries, including the United States, is greatly above the recommended value of 50 g/person/day (14–16). The mean dietary A:P protein ratio is around 65:35, with two thirds of the protein coming from meat, eggs and dairy. The Dietary Guidelines for Americans (DGA) are poised to reduce total protein, reduce amounts of meat, poultry and eggs, and increase the amounts of beans, peas and lentils in the USDA Healthy US Style Dietary Pattern (17, 18). While the Dietary Guidelines Advisory Committee report strongly favored plant-based diets (18), no specific targets for reducing dietary A:P protein ratios have yet been established.

By contrast, such targets are being promoted in the European Union. The EU protein strategy for human nutrition is built around plant-based proteins (11). Focusing on the need to reduce the A:P protein ratio in the French diet, the French National Institute for Agronomic Research and the Environment (INRAE) has suggested an A:P protein ratio of 50:50. At this time, the typical French diet derives up to 68% of protein from animal source foods (19). The Health Council of the Netherlands has proposed an even lower A:P ratio of 40:60 for consideration by the Dutch government (8, 20). In the current Dutch diet, the ratio is reversed (8). The Flemish Green Deal Protein Shift aims to shift the A:P protein ratio to 40:60 by year 2030 (21). Efforts to promote plant-based eating are also under way in Germany, Belgium, Sweden, and the United Kingdom (22–24). These efforts are driven by concerns about nutrition and health, the impact of agri-systems on the environment, and concerns with animal welfare (25–27). Plant-based diets are reputed to be associated with improved nutrition, greater affordability, and lower environmental footprint (28).

Local initiatives are also prominent. Twenty-five city governments globally have supported the 2021 Plant-Based Treaty in order to stem the impact of climate change, with Amsterdam becoming the first EU plant-based capital (29). The Plant Based Food Alliance in the UK has asked the current Labor government and other policymakers to help promote plant-based food consumption to achieve healthier and more sustainable diets (30). Dietary guidelines for the Netherlands, Germany and the United Kingdom refer to the need to limit the impact of existing dietary patterns on the environment. The European Green Deal has prioritized the production, provision, and consumption of alternative sources of proteins and has promoted dietary shifts toward sustainable plant forward diets (12). The Nordic Nutrition Recommendations feature a predominantly plant-based diet that is high in vegetables, fruits, berries, pulses, potatoes and whole grains (31). Denmark, Sweden and Germany were at one point considering a meat tax (32). Several companies have invested in alternative protein technologies to produce plant-based alternatives to meat, milk and dairy products (33).

On the global scale, the influential EAT-Lancet Planetary Health Diet has proposed an A:P protein ratio of approximately 30:70, with most of the dietary protein coming from grains, root crops, pulses, and from nuts and seeds (34). Beef and pork consumption in the Planetary Health Diet was restricted to only 7 g each per person per day, with higher amounts recommended for chicken and fish. Not sensitive to local traditions and cultures, the Planetary Health Diet has come under criticism for being inadequate in priority micronutrients (35) and unaffordable for the most part by the global poor (36).

The present objective was to assess the strength of the relation between country-level A:P protein ratios and gross national incomes (GNI). The main question was whether there were any countries that managed to combine high incomes with largely plant-based diets. The present approach was to use historical food balance sheets from Food and Agriculture Organization of the United Nations (FAO) (37) merged with gross national incomes (GNI) from the World Bank (38). Finding existing precedents might inform current efforts at reducing the proportion of animal proteins in the global diet.

One concern was that efforts by HIC actors to impose plant-based diets on a global scale may be in vain, since they run counter to the laws of economics (3, 4). Economic forces and diet structures appear to be inextricably linked, a phenomenon that gives rise to the well-established nutrition transition (3). As incomes rise, people eat less energy-dense root crops, legumes, and cereals and diversify their diets to include more animal-sourced foods and especially meat (3). The proportion of protein energy from root crops, legumes, and cereals declines whereas the proportion of protein energy from meats, eggs, and dairy increases. That fundamental observation is known as Bennett’s Law. Effectively, Bennett’s Law predicts that plant-based proteins will be replaced by animal proteins as an inevitable consequence of economic growth (3). It is a matter of record that the more affluent economies and wealthier consumers have more varied and higher-quality diets and seek out calories that are more expensive and more nutrient-rich (4).

## 2 Methods

FAOSTAT (37) food balance sheets for selected commodities (including animal and plant source foods) are used to calculate amounts of total protein, animal protein, and plant protein (in kg/capita/y) that are available for human consumption by country. The data are corrected for export and other food uses and apply to formal retail markets only. Informal markets are not included. FAO food balance sheets are supposed to be corrected for food waste and loss, including post-harvest losses, processing losses, distribution losses, and household and retail losses. The FAO uses global averages or specific country data to estimate waste and loss; however, accurate data can be lacking. The present analysis used FAOSTAT historical series estimates for energy from plant and animal proteins in calories/capita/day for the years 1961–2020 (37, 38). Despite their limitations, FAOSTAT data are routinely used as proxies for food consumption at country level. For example, diet modeling of the EAT Lancet Planetary Health Diet (39) relied on the FAO food balance sheets.

The World Bank classifies economies into four income groups: low, lower-middle, upper-middle, and high income. The approximate gross national income (GNI) categories, expressed in US dollars per capita are: low income (GNI < $1000), lower middle income ($1,000–$4,000), upper middle income ($4,000 to $13,000) and high income (>$13,000). The present analyses used historical GNI series for the years 2000–2020 (38). The World Bank incomes data are highly skewed and are conventionally presented following a log transformation. Historical data series spanning several decades are publicly available and can be downloaded from the FAO and World Bank websites, respectively. Multiple prior analyses confirming Bennett’s Law have used the same FAOSTAT food balance sheets, often joined with incomes from the World Bank (3).

## 3 Results

**Figure 1:**
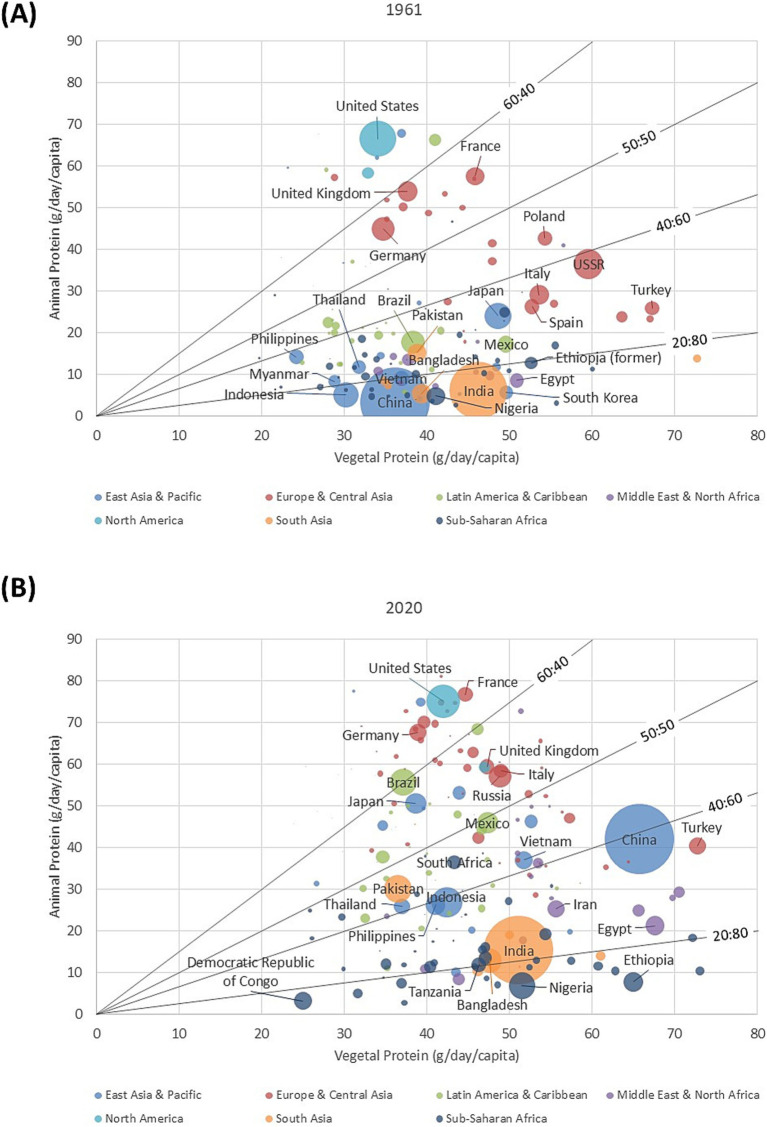
Animal protein (in g/day/capita) plotted against vegetal protein (in g/day/capita) from FAOSTAT by country in 1961 **(A)** and 2020 **(B)**. Countries are color coded by geographic region. The size of the circle reflects the country’s population size. Dietary A:P protein ratios are indicated.

Figure 1A shows the relation between animal protein in g/capita/day and vegetal protein also in g/capita/day that are available for human consumption by country. The data are from FAOSTAT historical series for 1961. Countries are color coded by geographic region and the size of the circle reflects population size by country. The dietary A:P protein ratios are indicated by black lines.

In 1961, the US was well above the dietary A:P protein ratio of 60:40. High income countries in Western Europe, France, Germany, and the UK had A:P protein ratios of between 50:50 and 60:40. In Eastern Europe, Poland and the USSR were well below the A:P protein ratio of 50:50. The two largest countries in Asia, India and China were below A:P ratio of 20:80 with relatively little animal protein in the diet. Countries in Africa had dietary A:P protein ratios that were even lower.

Between 1961 and 2020, total protein availability and the dietary A:P protein ratios increased substantially for most countries as shown in Figure 1B. The US, France, Germany and the Netherlands now all had dietary A:P protein ratios above 60:40. Reaching A:P protein ratio of 60:40 in the Americas was Brazil, with Mexico now close to the 50:50 line. In Asia, there was now a clear separation between China and India that could be cultural but could also be linked to sharp differences in the speed of economic growth during the intervening 60 years. China was now close to A:P protein ratio of 40:60, whereas India was still around the 20:80 line. Remaining below A:P protein ratio of 20:80 were some of the lower income countries in Africa, Ethiopia, Nigeria, and the Democratic Republic of the Congo.

**Figure 2:**
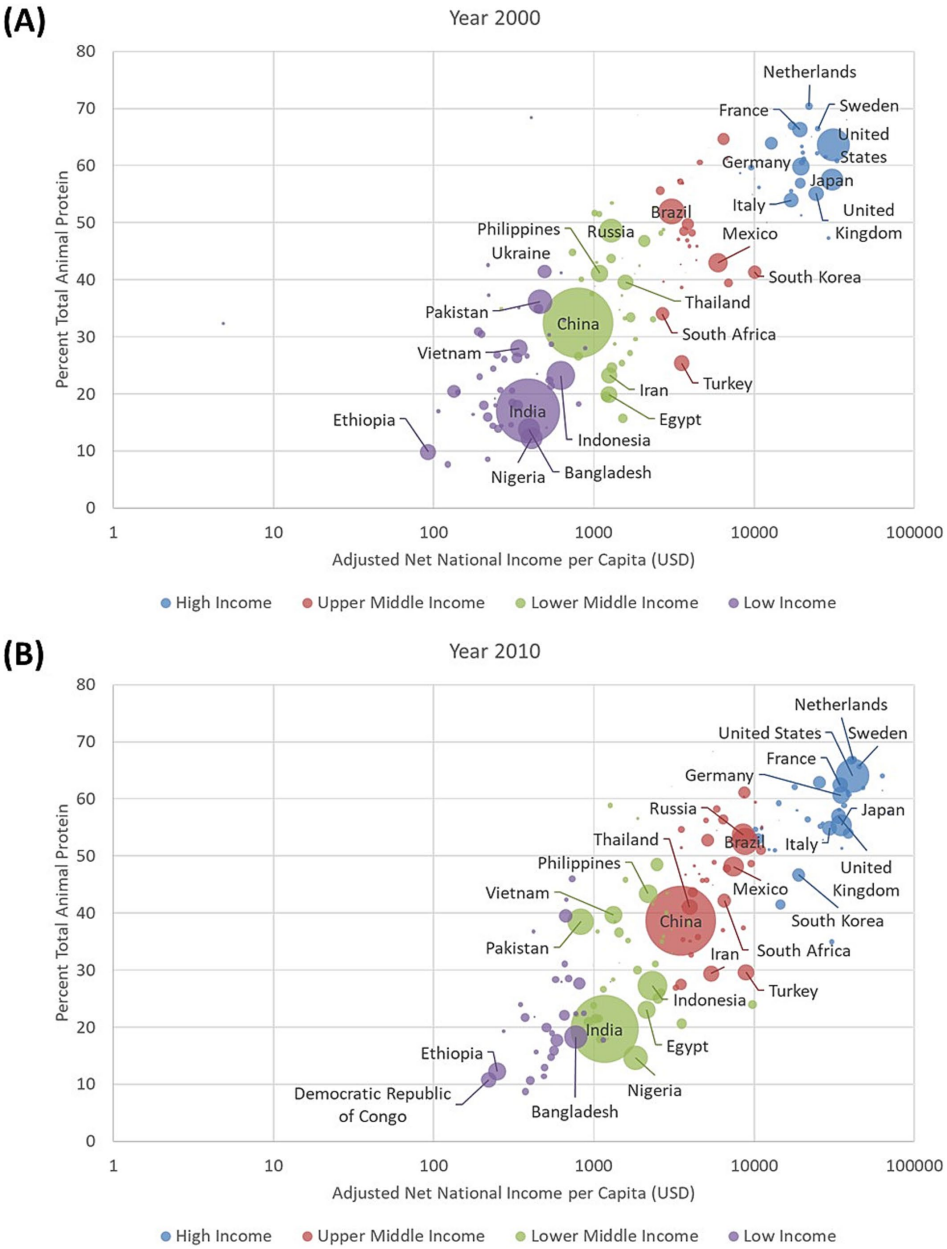
Adjusted net national income per capita in USD (on log-scale) plotted against the percentage of animal protein in the diet by country for year 2000 **(A)** and 2010 **(B)**. Countries are color coded by World Bank incomes categories. The size of circle reflects the country’s population size. Dietary A:P protein ration of 40:60 is indicated.

Figure 2A shows adjusted net national income per capita for 164 countries plotted on log scale against percent animal protein from FAOSTAT. The data are for year 2000. Country populations are indicated by size of the circle and the color coding reflects the World Bank income categories. The direct and strong relation between higher GNI and higher percent of animal protein (as percent of total protein) is very clear. Spearman correlation coefficient was 0.81. The reduced 40:60 A:P protein ratio that was proposed in the Netherlands is currently more characteristic of the Philippines, Thailand, and South Korea.

Figure 2B shows percent animal protein plotted against adjusted net GNI per capita for the year 2010. The strong relation between higher GNI and higher percent of animal protein (as percent of total protein) was unaltered. Spearman correlation coefficient was 0.81. Close to reaching the dietary A:P protein ratio of 40:60 were the rapidly developing Asian economies of China and Vietnam, in accordance with rising incomes. Remaining at around 20:80 A:P protein ratios were India and Bangladesh. Interestingly, in the Netherlands and in France there was evidence for a drop in percent animal protein between 2000 and 2010.

**Figure 3:**
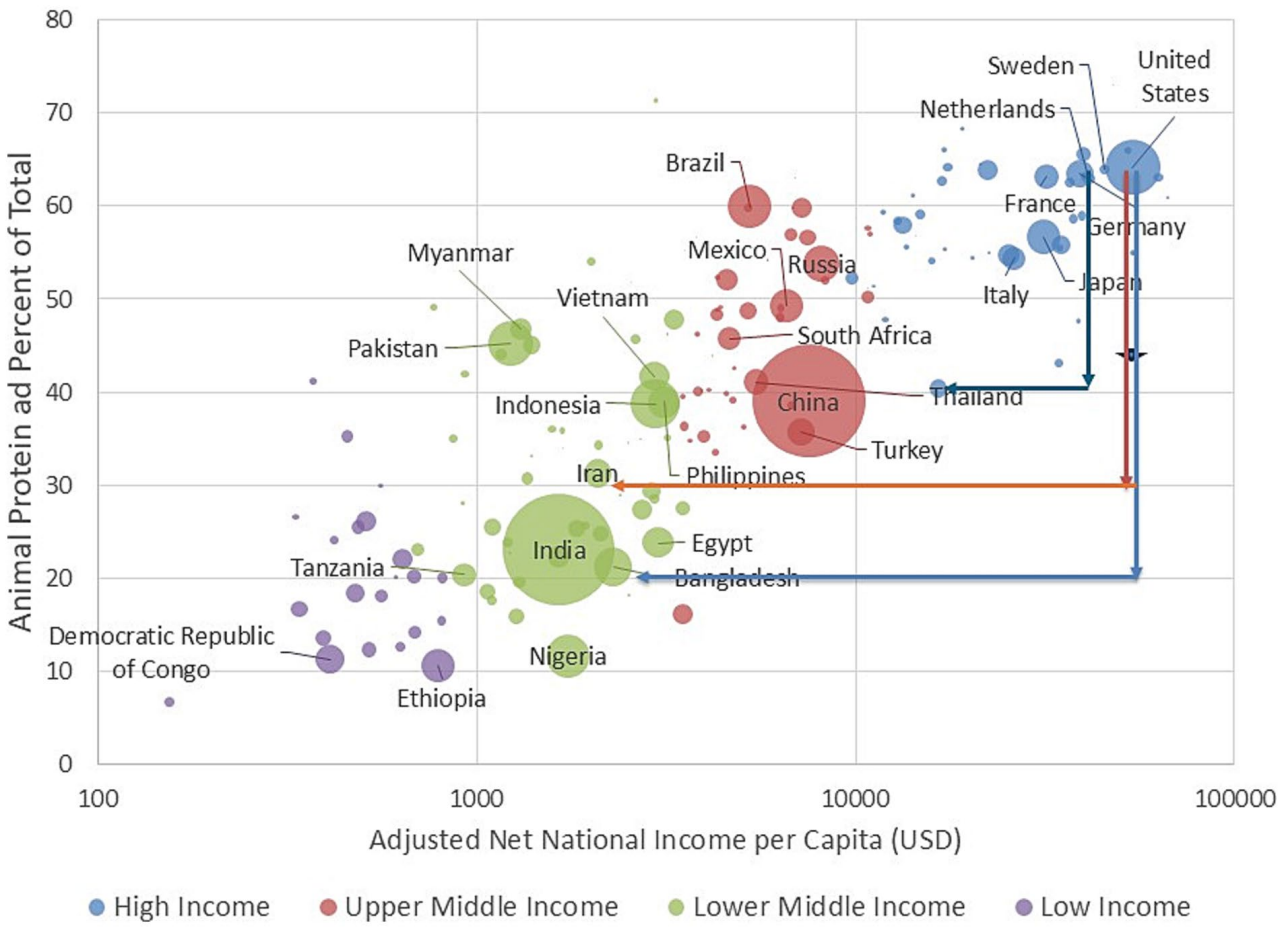
Adjusted net national income per capita in USD (on log-scale) plotted against animal protein as percent of total protein by country for 2020. Countries are color coded by World Bank incomes categories. The size of the circle reflects the country’s population size. Vertical arrows represent a change in A:P ratios at constant incomes. Horizontal arrows point to countries with specific dietary A:P protein ratios.

Figure 3 shows percent animal protein plotted against adjusted net national income per capita for the year 2020. Spearman correlation coefficient was 0.84. As shown before, China had A:P protein ratio of approximately 40:60, whereas India remained at around the 20:80 A:P protein ratio. Remaining below A:P protein ratio of 20:80 were lower income countries, mostly in Africa, with GNI values of below 1,000 USD per capita per year.

The dietary A:P protein ratios do not increase indefinitely with rising incomes and some slowing down is observed around 60% animal protein. No further increase was observed past GNI of 40,000 USD. At the highest country income levels, dietary A:P ratios were stable and higher incomes were no longer associated with higher dietary A:P ratios.

Figure 3 also shows likely pathways for reducing dietary A:P protein ratios by selected countries. For example, the Netherlands would most likely prefer to reduce the dietary A:P protein ratios at the population level without sacrificing incomes. That would be indicated by a vertical blue line in Figure 3. However, there are no existing precedents in the FAO data. Countries with the desired A;P protein ratio, as recommended by the Netherlands Health Council, are Thailand, China and Indonesia. Similarly, there are no historical or existing precedents for a country-level plant based diet for a country with incomes comparable to the US. Vertical lines going down to A:P ratios of 30:70 (EAT Lancet) or below show empty space and no precedents. Countries with dietary A:P ratios in that range are uniformly of much lower incomes, for example Iran and Bangladesh., as indicated by horizonal lines. Further down, dietary A:P protein ratios of 20:80 were characteristic of lower-income countries in Africa with least amounts of animal protein noted for Nigeria, Ethiopia and Democratic Republic of Congo.

**Figure 4:**
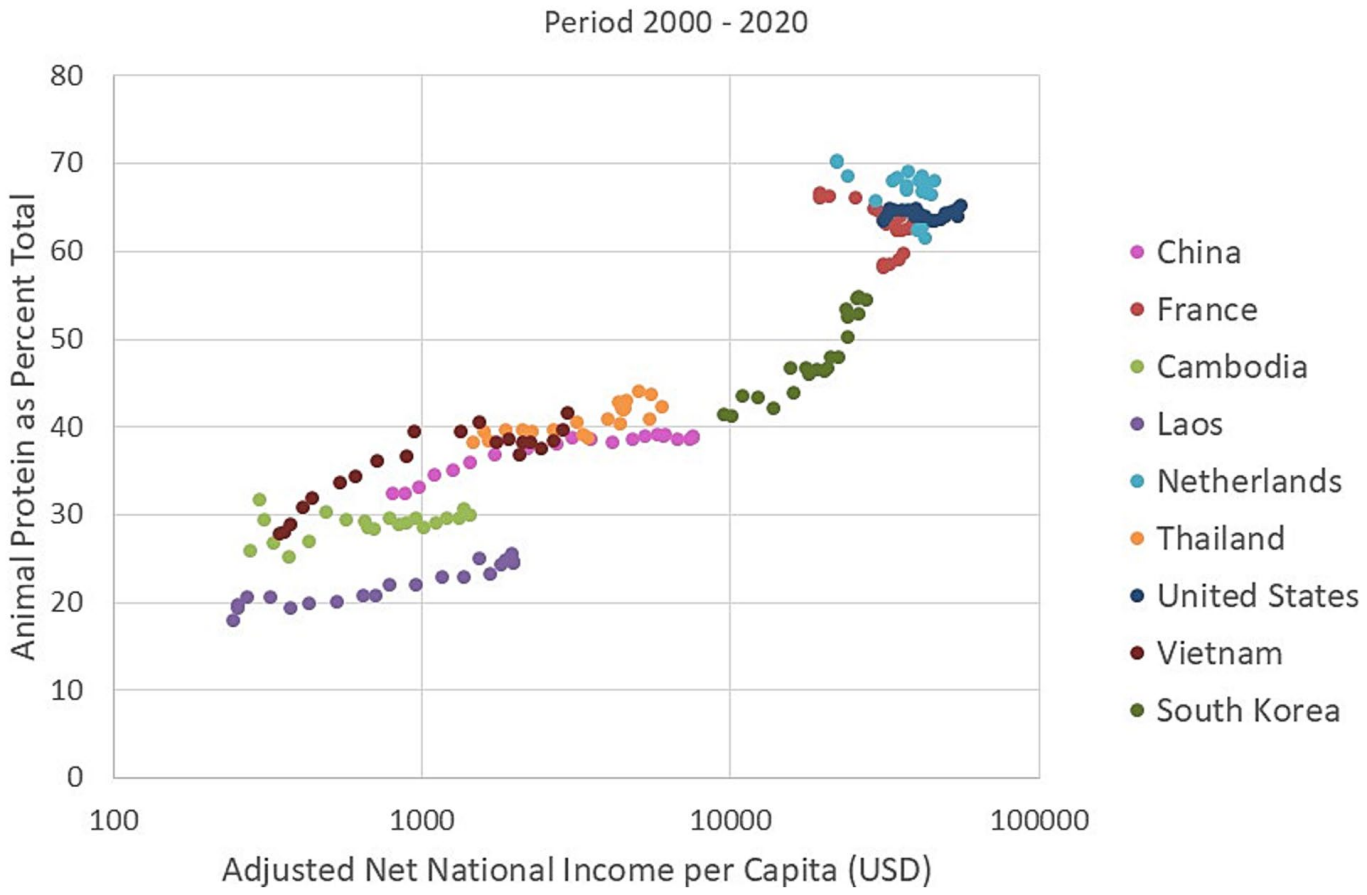
Temporal trends in percent animal protein (as percent total protein) from historical FAOSTAT data plotted against adjusted net national income per capita in United States dollars (USD) from The World Bank. The data for selected countries are for years 2000–2020.

Historical data permit analyses of time trends. Analyses of data for the period 2000–2020 confirm that rising incomes by country are associated with higher availability of animal protein, Figure 4 shows time trends for increases in percent animal protein for China, Indonesia, Thailand, Vietnam, and South Korea over the 20 year period. The growth was more pronounced for countries undergoing more rapid economic development, notably VietNam, China and South Korea. There was less economic growth for lower income Laos and Cambodia and the availability of animal protein did not rise.

It is interesting to note that the World Bank data point to little change in GNI values for France, the Netherlands and the US between 2000 and 2020. The corresponding FAOSTAT data for the same period point to no increase or even a decline in percent of animal protein available for human consumption.

## 4 Discussion

The present analysis, joining historical data series from the FAO and the World Bank, confirms the existence of a strong positive relation between GNI and the strong positive relation between GNI and the dietary A:P protein ratios. That relation between incomes and A:P protein ratios was little changed from 1961 to 2020 and held for each time point examined. Rising country-level GNI were associated with more total protein and with higher dietary A:P protein ratios. These observations confirm prior analyses (3, 40) and show the continued validity of Bennett’s Law.

According to most economic projections, and assuming rising incomes, the global demand for animal protein is likely to increase in the coming decades (41). Based on the sheer sizes of their populations, this increase will be driven by rising demand from the LMIC. About 85% of the global population live in lower-and middle-income countries. Only about 15% live in high-income countries, as defined by the World Bank. Even fewer live in those high-income countries that are currently promoting plant-forward diets on a global scale.

The LMIC protein transition is supported by local and regional governments. Traditional plant-based diets of starchy staples built around cassava, rice, and maize have been associated with inadequate intakes of priority micronutrients iron, zinc, calcium, vitamin A (retinol), vitamin B12, and vitamin D (42). Plant-based proteins can lack lysine and have been associated with amino acid imbalance. Some of these deficiencies can be remedied by the addition of small amounts of animal-source foods to the diet (42, 43). Such foods provide bioavailable iron, zinc, calcium, vitamins A (retinol), B12, and other B vitamins in addition to high-quality protein (44). Incorporating *more* meat in the diet is one way to address micronutrient inadequacies, still prevalent across the LMIC. It is worth noting that local production of animal foods, livestock and dairy, has been increasing in Africa and Asia, with support from international agencies, notably FAO (6). Interestingly, the rising global demand for meat favors chicken and pork, rather than the more costly beef (3, 40).

It is important to note that the protein transition does not occur in all countries at the same time or at the same speed (1). Further, social as well as economic factors are involved (1, 5, 25). For example, in South Korea, rapid economic development was associated with a sharply higher meat consumption, notably pork. Farther down the economic scale, India has stayed with the dietary A:P protein ratio of about 20:80, most likely due to local religion and culture and despite significant economic growth. Major differences in protein consumption patterns have also been observed between Indonesia and Malaysia (5), countries that share the same geographic region but are very distinct in terms of economies, populations, traditions, and cultures.

Further analyses of FAO data indicate that some highest income countries may have reached peak meat consumption (3, 45). We confirm prior observations (based on the same FAO and WB data) that the dietary A:P protein ratio fails to increase further above GNI of approximately $40,000. For some countries (notably France and the Netherlands) there were suggestions of a decline. Indeed, data from the French Ministry of Agriculture show that per capita meat consumption fell by 5.8% from 2003 to 2023, reaching 83.5 kg (46). Beef was replaced by lower-cost chicken.

Descriptions of the “healthy protein transition” in high-income countries rarely mention the influence of economics on consumer behavior (47, 48). Based on scoping literature reviews, the three main pathways to reducing dietary A:P protein ratios have been identified as consumer education and behavior change, technological advances in the manufacture of alternative proteins, and associated government-led policy, taxation, and regulatory measures (2). All of these seem to require top-down government interventions and can be viewed as more or less coercive (2). There is recognition that a comprehensive, if not compulsory, restructuring of food systems will be needed as well. For example, the Netherlands Organization for Scientific Research (NWO) recognizes that speeding the transition to plant-based proteins (20) will require major shifts in crop and animal production systems (24). Publicized EU policy measures have stressed the need to transition agri-food systems from “an animal-dominated regime to an alternative protein regime,” which, according to some sources, could potentially mean the end of livestock farming in the EU (49). Other research studies, with varying degrees of emphasis, propose to disrupt existing EU food systems, stop the “meatification” of global diets, and end the influence of “Big Meat” (50).

The attempts at coercion and/or disruption have met with mixed success. The consumption of meat and dairy is so deeply rooted in European culture that replacing them with manufactured plant-based alternatives will not be easy (51). Proposed legislative measures to buy out and shut down livestock farms in the Netherlands had political repercussions (49, 52). In November 2023, the Party for Freedom won the general election, leading to a shift in policy direction. Attempts by the Mayor of Paris to institute vegan Paris Olympics met with general derision (53). When it comes to technological advances, ultra-processed plant-based meat alternatives have not met with the expected commercial success. Several nations are proposing measures to limit the use of meat and dairy terms to describe manufactured plant-based alternatives (54). However, there is potential for more plant protein from more traditional sources, namely pulses, legumes, nuts and seeds.

It is concerning that the growing literature on the sustainability benefits of plant-based diets is generated *exclusively* in high-income countries. Consumers in, e.g., Nigeria, living on $1 per day, may not have the same concerns with the environment as consumers in Sweden. Similarly, the health benefits of plant-based diets seem to apply to high income countries only (2). The fact that an opposing protein transition is taking place globally was barely mentioned in a scoping review (2). That might explain the current belief across some EU countries that the fundamental relation between economic forces and diet structures can be uncoupled at will.

Two fundamental questions need to be asked. First, should low-income countries abandon their aspirations to a healthier, more varied, and more nutrient-rich diet? Rich-country researchers are already thinking of ways to encourage LMIC populations to maintain their traditional “healthy” eating patterns (25). In particular, the traditional Latin American, Asian, and African “heritage” diets, previously associated with malnutrition, are now being touted as cultural models of healthy eating (55).

Viewed in this context, efforts by rich country actors to reduce dietary A:P protein ratios on a global scale, as exemplified by the EAT-Lancet Planetary Health Diet (34) appear to run counter to the laws of economics, namely Bennett’s Law. The present analysis of global diet structures suggests that efforts to impose a single planetary health diet will most likely fail, as perhaps they should.

Second, there is little precedent for high-income countries to follow a largely plant-based diet on a population basis. Such diets do exist as demonstrated by FAO data. It is the feasibility of their adoption by high income countries that is unclear. In other words, will the population of France willingly adopt a diet with a 50:50 A:P ratio that is more characteristic of Mexico? Will the Netherlands, in a reversal of its colonial past, adopt a diet with a 40:60 A:P ratio that is more characteristic of today’s Indonesia? Will the higher-income countries voluntarily adopt the EAT-Lancet Planetary Health Diet with and A:P ratio of 30:70? The present concern is that, barring some economic calamity, they will not.

As food prices increase or incomes drop, HIC consumers have been observed to trade down to cheaper types of meat, buy less meat, or adopt flexitarian diets with more grains, pulses and beans (3). Studies have linked recent adverse economic events to a lower consumption of animal proteins. For example, the consumption of meat and fish decreased during the great recession of 2008, with meat replaced by more eggs and plant based proteins (56–59). Faced with lower incomes, consumers ate less meat and more proteins from pulses and grains (3). In another study of purchase preferences in Finland, plant-based proteins were associated with lowest household incomes (60). In other words, the ostensible desire for more plant proteins may have been driven by the failing purchasing power and by rising prices for meat, poultry, and fish.

## 5 Conclusion

The present analyses points to a consistent and strong relation between rising incomes and more animal protein available for human consumption. Other than for a handful of the richest countries, this relation is predicted by Bennett’s Law. That falling incomes are associated with less animal protein can be viewed as a corollary to Bennett’s Law. In other words, stagnating wages and lower incomes, rather than consumer education, may drive consumers inexorably toward more plant-based foods. It is tempting to interpret some of the current efforts at reducing the dietary A:P protein ratios in the European Union, ostensibly driven by concerns with health and the environment, as mere manifestations of the fading economic power of Western societies.

## Data Availability

Publicly available datasets were analyzed in this study. This data can be found at: FAOSTAT data repository of the Food and Agriculture Organization of the United Nations, https://www.fao.org/faostat/en/#home. World Bank data repository on adjusted net national incomes per capita, https://data.worldbank.org/indicator/NY.ADJ.NNTY.PC.CD.
